# Diet, Exercise, and the Metabolic Syndrome: Enrollment of the Mitochondrial Machinery

**DOI:** 10.3390/nu14214519

**Published:** 2022-10-27

**Authors:** Elena Silvestri, Antonia Giacco

**Affiliations:** Department of Science and Technology, University of Sannio, Via De Sanctis, 82100 Benevento, Italy

Metabolic syndrome (MS), a cluster of metabolic risk factors, ranging from abdominal obesity, dyslipidaemia, hypertension, type 2 diabetes and non-alcoholic fatty liver disease [1], is one of the major global health problems due to its growing incidence and prevalence [2]. Although the topic is the subject of careful research studies, the underlying etiology is still not fully understood. Many contributing factors and mechanisms have been proposed including insulin resistance (IR), adipose tissue dysfunction, chronic inflammation, oxidative stress, alterations of the gut microbiota and, to a lesser extent, genetic factors [3,4,5,6,7]. However, environmental and lifestyle factors such as the consumption of excess calories and a lack of physical activity have been characterized as being major contributors. Visceral adiposity paves the way for the primary trigger for most of the pathological features of MS, thus stressing the impact of sedentarism and over nutrition [8].

Therapeutic approaches to MS emphasize lifestyle changes including dietary interventions associated or not associated with physical activity, especially to reduce body weight and the associated risk of cardio-metabolic diseases. Notwithstanding the well documented beneficial effects of caloric restriction regiments in reducing body weight and improving body composition in both humans and animals [9,10,11,12], research attention has been recently focused on the usage of the “healthy diets” over the simple restriction of calories. There are numerous examples of such dietetic regimes: the Dietary Approaches to Stop Hypertension (DASH) diet, low carbohydrate-diet, low fat diets, plant-based diets, and the classic Mediterranean diet, the latter being beneficial as it is paradoxically classified as a high-fat diet [13]. This definition, however, is less paradoxical than it may seem when taking into account that the Mediterranean diet involves the almost exclusive consumption of extra virgin olive oil as a seasoning fat and is rich in oleic acid and several other minor components, which, together with a high quantity of polyphenols, exert actual therapeutic effects. Indeed, several studies have revealed how the Mediterranean diet reduces the risk of developing MS independent from age, gender, and physical activity in healthy subjects and in MS subjects as well [14,15].

Together with dietary interventions, exercise therapy helps to both prevent and mitigate the impact of MS. Chronic resistance and endurance exercise training, alone or in combination, reduce body weight, blood pressure, and improve lipid profiles, e.g., raising high-density lipoproteins (HDL) and lowering triglycerides [16,17,18,19,20]. The most beneficial effects of exercise training have been reported on IR [20,21,22,23]. Specifically, there is evidence that vigorous exercise not only reduces adipose tissue mass but also induces the browning of white adipose tissue, enhancing glucose and lipid metabolism, finally resulting in improved insulin sensitivity [23].

This Special Issue of Nutrients, “Diet, Exercise, and the Metabolic Syndrome: Enrollment of the Mitochondrial Machinery” aimed to further elucidate the biochemical and molecular links between metabolic disturbances, mitochondrial structural/functional changes, and lifestyle interventions which still remain not completely understood and urgently need further investigation.

The Special Issue collected three original articles on the topic. These articles allow the reader to focus on whether and how diet, exercise and bioactive compounds impact whole body metabolism and the mitochondrial compartment in metabolically relevant tissues. In particular, the results presented and discussed in the three articles allow the following questions to be answered:

Can resistance exercise reverse diet-induced obesity-related deleterious metabolic and inflammatory effects?

What are the molecular and biochemical factors involved in the beneficial effects of mild endurance exercise in settings of energy restriction?

Can alterations of polyunsaturated fatty acids (PUFA) metabolism impact mitochondrial functions?

Pinho and co-workers, with the aim to investigate whether resistance exercise can reverse whole-body and skeletal muscle obesity-induced deleterious metabolic and inflammatory effects, used a new training protocol, consisting of ladder climbing, rarely applied in rodents, to resemble the typical resistance exercise in humans [24]. This protocol applied in mice high fat diet- obese, insulin-resistant and with alteration of redox balance in skeletal muscle, resulted to be effective in reducing adiposity, ameliorating whole body glycemic control and, at the skeletal muscle level, in reducing the content of inflammatory mediators [tumor necrosis factor-alpha (TNF-α) and interleukin 1 beta (IL1-β)] while increasing the phosphorylation of AktSer473 and AMPKThr17 [24]. These results furnish new details on the molecular players involved in the beneficial effects of resistance exercise and encourage the elaboration of new exercise approaches to translate into humans as a therapy against obesity and associated diseases.

The beneficial effects of physical activity go beyond skeletal muscles and involve several adaptations in other organs [25]. In the brain, exercise promotes different physiologic phenomena, including angiogenesis, neurogenesis, and synaptogenesis [26]. From both human and animal studies, it has emerged that most of the protective mechanisms of the brain induced by physical exercise derive by the stimulation and release of the neurotrophic factors, among which there is brain-derived neurotrophic factor (BDNF) [27,28,29]. Indeed, physical exercise was shown to be effective in enhancing circulating levels of BDNF and improving brain function [27]. Similar effects have been obtained under conditions of caloric restriction [30,31]. Additionally, the combination of both stimuli also increases BDNF expression in skeletal muscles with systemic beneficial effects [9].

De Lange and co-workers tested a very mild endurance exercise protocol on rats which resulted in positively affecting the brain and skeletal muscles under conditions of energy restriction involving, precisely, BDNF, mammalian target of rapamycin (mTOR) activation and thyroid hormone (T3) [32]. More specifically, BDNF-CREB-mTOR pathway activation is associated with the normalization of skeletal muscle and brain cortex intratissutal level of T3, normally decreased during fasting, and the modulation of peripheral deiodinase expression. These results highlight important implications on health, with it being universally known that T3 not only in itself acts as a mimetic of physical exercise, but also elicits a pleiotropic effect on whole body metabolism and on brain function, likewise for cognition, memory learning and behavior [33,34,35]. Nevertheless, physical activity as a treatment for metabolic disease remains underestimated, whereas pharmacologic treatments or other interventions which are more economically driven are preferred, especially in the advanced state of MS-related diseases.

Looking inside the cells, an increasing number of studies indicate that the oxidative stress condition strongly contributes to trigger the metabolic diseases with the derangement of the mitochondrial compartment. Mitochondria are the powerhouses of the cell and play a key role in maintaining homeostasis by finely regulating the balance between energy storage and energy expenditure. Their principal function is to synthesize ATP via the oxidative phosphorylation system (OXPHOS) maintained by the oxidation of metabolites through the Krebs cycle and β-oxidation. Moreover, mitochondria represent the main source of cellular ROS. An estimate of about up to 2 percent oxygen consumed can be deviated to the ROS formation [36].

High energy diets increase the flux of substrates to mitochondria, resulting in over activation of OXPHOS that can form excessive ROS as by-products. The ROS, in turn, can directly impair the mitochondrial compartment itself, affecting the cellular redox signalling. Indeed, ROS have been associated in the first place with oxidative damage on lipids, DNA, and proteins [36]. Over the time, oxidative damage conditions can lead to chronic inflammation triggering metabolic disorders [36,37,38].

The evidence of the involvement of mitochondrial dysfunction and related stress pathways has opened the way to research for potential therapeutic purposes in metabolic disorders targeting these organelles [39].

Genetically modified models have been fundamental in understanding the best interventions to improve mitochondrial functionality and health. Studies in both animals and humans have show the ameliorating effects of antioxidants on mitochondrial function in the presence or absence of metabolic disorders [40,41]. For example, the commonly used vitamins and other chemical compounds with antioxidant properties may help to reduce ROS accumulation in several dysfunctional metabolic contests [42]. Moreover, direct targeted mitochondrial compounds have been successively used against mitochondrial damage [36]. Physical exercise training in itself may help to reduce mitochondrial damage, although it is influenced by the different cellular contests. Indeed, excessive physical loading may generate detrimental effects by increasing pro-oxidant species. In contrast, regular habitual physical exercise increases metabolic flexibility, by influencing cell fuel utilization or directly mitochondrial network. In light of this, exercise training regimens can be considered as a therapeutic-like intervention targeting mitochondria in several tissues. In skeletal muscles, exercise can preserve the quality of the mitochondrial network [43]. Both endurance-based and resistance-based exercises promote health benefits by increasing mitochondrial content and function in skeletal muscle [44].

Among healthy nutrients and foods, it is crucial to pay attention to the so defined “functional foods”, which contain biologically active molecules associated with physiological health benefits for preventing and managing chronic diseases [45]. In recent times, phenolic compounds have shown the ability to prevent some chronic and degenerative diseases, likewise cardiovascular diseases, type 2 diabetes, some types of cancers or neurodegenerative disorders. Ginkgo biloba extract (GBE), resveratrol, and phytoestrogens as well have shown not only some mitochondria-modulating properties but also significant antioxidant potential in in vitro and in vivo studies [46]. There is strong evidence that the beneficial effects of resveratrol are carried out through the protection of mitochondrial function and the activation of biogenesis, directly targeting mitochondria [47]. Fatty acids contained in the food may have different effects on health according to their saturation grade bonds [48]. An increasing number of studies have shown that n-3 polyunsaturated fatty acids (n-3 PUFAs), including eicosapentaenoic acid (EPA, 20:5 n-3) and docosahexaenoic acid (DHA, 22:6 n-3) exert beneficial effects on metabolic diseases, likewise IR, cardiovascular diseases, and inflammation-associated diseases [49]. These beneficial effects are attributed to the reduction of mitochondrial dysfunction by stimulating β-oxidation and inhibiting lipogenesis. However, some discrepancies between animal and human studies preclude any practical application and prompt further studies for the use of n-3 PUFA as nutritional therapies in the prevention of IR in humans [50]. Another warm and poorly investigated topic of discussion concerns the different impact on health and mitochondrial functionality of PUFA taken up from diet versus those produced endogenously.

Elongation of very long chain fatty acids protein 2 (ELOVL2)—ablated mice offered the possibility to investigate such issues, considering that the ELOV2 gene controls the endogenous PUFA synthesis. Indeed, Elovl2 KO mice display, in liver and serum, substantially decreased levels of omega-3 [e.g., docosahexaenoic acid (DHA)] and omega-6 [docosapentaenoic acid (DPAn-6)] [51], which are associated with the remodeling of the phospholipid composition of the mitochondrial membranes. Nevertheless, DHA is required not only for normal brain development in children, but also for brain function in adults [52].

Shabalina and coworkers collected evidence about the importance of endogenous long-chain PUFA production for proper mammalian mitochondrial function and metabolism. In the liver, the absence of the Elovl2 enzyme in the endoplasmic reticulum drastically reduced the content of mitochondrial DHA (and of other PUFA) and this was in parallel associated with a decrease in mitochondrial respiration efficiency despite the absence of oxidative damage and preserved content of proteins of the respiratory chain [53]. Although most PUFA derive from dietary intake, it is clear that endogenous production is very important for mitochondrial functionality maintenance.

On the basis of the current literature, it is clear that much research has been carried out and now more is known about the effects of diet and exercise on human health, however the pleiotropic effects of lifestyle interventions prompt us to shed further light on a topic of such sensitive interest for public health. Overall, the original contributions included in this Special Issue are examples of new studies that are able to increase knowledge in the field and contribute to the open debate on whether and how healthy diet and exercise are useful approaches to prevent and/or counteract metabolic diseases, with emphasis on the impacts of dietary composition, feeding frequency, exercise training, and bioactive compounds on the mitochondrial compartment. What is expected is that this Special Issue will give new support to translational interventions in the specific targeting of metabolic diseases in the broad spectrum of pathological conditions which generate MS.
